# Neuropharmacology Potential of the Hydroalcoholic Extract from the Leaves of *Piper cernuum*: Anxiolytic, Hypnotic, and Antidepressant-Like Effects

**DOI:** 10.1155/2023/1183809

**Published:** 2023-04-10

**Authors:** Marcel Andrigo Maia, Jocilene Demétrio Jurcevic, Angela Malheiros, Camila André Cazarin, Ana Paula Dalmagro, Camila do Espírito Santo, Luisa Mota da Silva, Márcia Maria de Souza

**Affiliations:** ^1^Postgraduate Program in Pharmaceutical Sciences, University of Vale do Itajaí, Itajaí, SC, Brazil; ^2^Nucleus of Chemical-PharmaceuticalResearch-NIQFAR, University of Vale do Itajaí, Itajaí, SC, Brazil

## Abstract

**Aim:**

The use of medicinal plants in the treatment of mental illnesses is a reality that accompanies the history of civilizations, and the Piper genus exhibits many species with pharmacologically proven central effects. Then, this study evaluated the neuropharmacological effects of the hydroalcoholic extract from *Piper cernuum* (HEPC) leaves to validate its uses in folk medicine.

**Materials and Methods:**

Primarily Swiss mice (female, 25–30 g) were pretreated with HEPC (50–150 mg/kg, p.o.), vehicle, or the positive control, and submitted to open-field test (OFT), inhibitory avoidance test (IAT), tail suspension test (TST), and forced swim test (FST). Also, mice were exposed to pentylenetetrazol- and strychnine-induced seizure assay, pentobarbital-induced hypnosis test, and elevated plus-maze (EPM). The GABA levels and MAO-A activity were measured in the animal's brain after 15 days of HEPC administration (150 mg/kg, p.o.).

**Results:**

Mice pretreated with HEPC (100 and 150 mg/kg) and exposed to pentobarbital presented decreased sleep latency and increased sleep duration (HEPC 150 mg/kg). In EPM, the HEPC (150 mg/kg) increased the frequency of entry and the time of exploration of mice in the open arms. The antidepressant-like properties of HEPC were demonstrated by the decrease in the mice's immobility time when tested in FST and TST. The extract did not show anticonvulsant activity, in addition to not improving the memory parameters of animals (IAT) or interfering with their locomotor activity (OFT). Besides, HEPC administration decreased the MAO-A activity and increased the GABA levels in the animal's brain.

**Conclusion:**

HEPC induces sedative-hypnotic, anxiolytic-, and antidepressant-like effects. These neuropharmacological effects of HEPC could be, at least in part, related to the modulation of the GABAergic system and/or MAO-A activity.

## 1. Introduction

Anxiety, depression, and epilepsy are neurological disorders with significant social impacts especially nowadays [1, 2]. Associated with these diseases are also sleep disturbances that constitute not only a symptom of such pathologies but can also be characterized as a pathology other than [3]. Moreover, epilepsy is a severe neurological disorder with a prevalence of 6.38 per 1000 persons worldwide [4].

Recent data estimate that about 4.4% of the world's population, approximately 322 million people live with depression, while the estimated number of patients with anxiety in the world is about 264 million [5]. For such diseases, current treatments vary in effectiveness considering individual differences, which can lead to cases of refractoriness [6].

It is known that from ancient times, people have used different methods and procedures to treat psychiatric disorders and have often used medicinal plants as one of these resources [7–9]. Indeed, medicinal plants are important sources of biologically active substances, providing the basis for pharmacological research and discovering new molecules with antidepressant, anxiolytic, hypnotic, and anticonvulsant effects [10, 11]. Considering this background, it is necessary to provide scientific information about the pharmacological profile of medicinal plants to identify their safety and efficacy [12], collaborating to validate their uses.

One widely distributed plant in pantropical regions is the *Piper* genus. *Piper* plants are also known under the common name “pepper.” The presence of oil cells in the structures of almost all *Piper* sp. places them in the group of aromatic plants [13]. Besides their well-known uses as culinary spices, the secondary metabolites isolated from these plants, among which are terpenoids, lignans, and alkaloids, among other classes, show wide-ranging human health effects [10]. Regarding psychopharmacological properties, the effects of Kava (*Piper methysticum*) are already known, which has been explored as a potential phytotherapy option for generalized anxiety disorder [14]. Furthermore, the pharmacological potential of *Piper nigrum* as a neuroprotector against epilepsy and biochemical alterations arising from Alzheimer's Disease is also reported in the literature [15, 16].

Other *Piper* species are underexplored for their pharmacological properties despite widespread uses, and an example is *Piper cernuum* Vell, popularly known as “Pariparoba,” “João-guarandi-do-grado,” and “Pimenta-de-Morcego.” This species is considered a medicinal plant and is used by rural communities to treat pain conditions, such as bellyache and muscle pain (topically), besides hepatic and renal complications [17].

There is little scientific information about the *P. cernuum* leaves extract effects but are data showing its antileishmanial [18] and cytotoxicity effects against oral squamous cell carcinoma effects [19]. In addition, from leaves extracts the following cinnamic acid derivatives were isolated: methyl 3,4-dimethoxy-dihydrocinnamate, 3,4-dimethoxy-dihydrocinnamic acid, methyl 3,5-dimethoxy-4-hydroxy-dihydrocinnamate (methyl dihydro sinapate), methyl 3,5-dimethoxy-4-hydroxy-cinnamate (methyl sinapate), and cubebin [20]. (−)-Bornyl p-coumarate too isolated from leaves exhibited antitrypanosomal activity and effect on plasma membrane permeability [21]. However, the essential oils of this species have already been studied in relationship to antimicrobial activity and chemical composition [22–28]. Some compounds present in oil exhibit antituberculosis [29] and antitumor [30] effects too.

Recent results obtained in our laboratories indicated that the hydroalcoholic extract of *P. cernuum* leaves showed no toxicity when administered to male or female rats, lower cytotoxicity, and mutagenicity [31]. Therefore, these previous reports about its safety and the neuropharmacological potential of species of this genus led us to experimentally investigate whether the hydroalcoholic extract of *P. cernuum* leaves has psychopharmacological effects like other species of the genus currently identified as potent natural sources and alternatives for the treatment of human illness, especially mental illness.

## 2. Material and Methods

### 2.1. Plant Material and Extract Procedure

*P. cernuum* Vell leaves were collected (a cultivated specie) in Blumenau, a city in the state of Santa Catarina, Brazil (latitude: 26°58′43.8″S, longitude: 49°03′43.0″W, and altitude: 200 m) in March in the years of 2015. Prof. Andre Luis de Gasper, Curator of the Dr. Roberto Miguel Klein Herbarium from the Regional University of Blumenau, classified the material. Samples of the specimen were deposited in this Herbarium with the number 41606. This specie was registered on National System for the Management of Genetic Heritage and Associated Traditional Knowledge (SisGen) under registration number A862470.

The extracting procedure was described by Wolff et al. [31]. The *Piper cernuum* leaves were dried at 40°C for seven days after the material was triturated and submitted to agitation at Bertel agitator. The particles with diameters between 0.31 e 0. and 57 mm were used. The procedure of extraction was conducted under dynamic maceration at room temperature for 4 hours. Plant material (250 g) was successively extracted with ethanol 90°GL, plant: solvent ratio of 1 : 10 (w/v). The extractive solution was filtered under vacuum filtration after it was concentrated under reduced pressure and finally the extract was dried under a current of air at 50°C giving a yield of 5.7% in relation to leaves. The phytochemical profile of the hydroalcoholic extract from *Piper cernuum* (HEPC) was already described by Wolff et al. [31].

### 2.2. Pharmacological Tests

#### 2.2.1. Animals and Ethics

Female Swiss mice (25–35 g) were obtained from Central Animal Facility/UNIVALI and maintained at 22–24°C with water and food *ad libitum*, under a 12 : 12 h light/dark cycle (lights on at 07 : 00 h). Female mice were employed in this study considering the higher prevalence of depression in women, as well as the greater availability in the vivarium. In addition, females with a controlled estrous cycle were used to avoid interference in the results. All procedures were conducted between 9:00 a.m. and 04:00 p.m. and each animal was tested only once. Behavioral tests were performed in real-time by trained researchers, taking care to minimally stress the animals. All efforts were made to minimize animal suffering and to reduce the number of animals used in our experiments, and the procedures were performed according to the Brazilian Society of Science of Laboratory Animals' guidelines for the proper care and use of experimental animals. The experimental procedures were approved by the Ethics Committee of the University of Vale do Itajaí, Brazil (protocol no. 037/2019).

#### 2.2.2. Drugs and Treatments

Several drugs used in the research were obtained from Sigma Chemical Co. (St. Louis, USA): pentylenetetrazol, strychnine, sodium pentobarbital, and imipramine. Diazepam was obtained from Cristália (SP, Brazil). Each drug was dissolved in saline (0.9% NaCl) and administered by intraperitoneal (i.p.) route in a volume of 10 mL/kg body weight. HEPC (50, 100, 150 mg/kg), solutions were prepared daily with distilled water plus 2% DMSO before the use and administered orally (p.o.) by gavage in a volume of 10 mL/kg. The vehicle (negative control) consisted of distilled water plus 2% DMSO and was administered orally (p.o.) by gavage in a volume of 10 mL/kg. All treatments with HEPC and vehicle were performed 1 hour before the experiments. The experimental N was 06–08 individuals per experimental group.

#### 2.2.3. Open-Field Test (OFT)

The first behavioral test used in this study was the OFT. Depending on the protocol adopted, many studies on drug effects usually employ the OFT to evaluate locomotor and exploratory parameters of animals before submitting them to tests to verify anxiolytic-like and antidepressant effects [32]. The open-field apparatus consisted of a 40 × 60 cm dark plastic box with 50 cm high boundary walls, and the box was divided into 12 equal squares. After treatments, vehicle, and HEPC (50, 100, and 150 mg/kg, p.o.), the animals were individually placed in the center of the box for 30 s for adaptation and then allowed to freely explore the area for 6 min. The animal's locomotor activity was registered as the total number of times (counts) that the animal crossed a square during the test. A count was considered when the animal crossed from one square to the next. The open-field test was performed at 30 (for intraperitoneal treatments) or 60 (for oral treatments) min after drugs or extract treatments. Locomotor activity was defined as a horizontal activity with displacement and was expressed in terms of the total number of quadrantes crossed. In this test, the number of rearing performed by each animal was also recorded. Rearing was characterized by the behavior of the animal to lift the body and keep it upright, leaning on its hind legs to explore the apparatus. After each trial, the apparatus was cleaned with 70% ethanol to remove the odor clues left by the previous animal.

#### 2.2.4. Pentylenetetrazol (PTZ) and Strychnine (STR) Induced Seizure Test

The anticonvulsive activity of the plant extract was evaluated through the pentylenetetrazol (PTZ) and strychnine (STR) tests. These tests were based on the method described by Holzmann et al. [33] with slight modifications. In the first experiment, 60 minutes after the oral administration of vehicle or HEPC (50, 100, and 150 mg/kg, p.o.), PTZ (100 mg/kg, i.p.) was administered individually in mice, and immediately the animals were transferred to the observation box. The documented parameters in this experiment were latency to seizure-onset and the number of mice not exhibiting seizures. The seizure was defined as jerky movements of the whole body or convulsion. Each mouse was observed for one whole hour for the occurrence of seizure. The same procedure was performed in another set of experiments; however, the convulsant agent utilized was STR (4 mg/kg). Phenobarbital (30 mg/kg, i,p), the positive control used in both experiments, was administered 30 min before testing.

#### 2.2.5. Pentobarbital-Induced Hypnosis

To evaluate the hypnosis-potentiating activity of the extract, sodium pentobarbital (50 mg/kg, i.p.) was injected into female mice 60 min after oral administration of HEPC (50, 100, and 150 mg/kg). The latency and hypnosis time were recorded. Hypnosis time was measured by the loss of the righting reflex, with the recovery of this reflex being considered the end of the hypnosis time [33]. Diazepam (2.0 mg/kg, i.p.) was administered 30 min before the test as a reference drug.

#### 2.2.6. Elevated Plus-Maze Test (EPM)

The procedure used to evaluate the possible anxiolytic effect was like that described previously [32]. The wooden EPM apparatus was shaped as a cross, with two open arms (50 × 10 cm^2^) with sides of 1 cm in height, and two closed arms (50 × 10 × 15 cm). The central area of the maze measured 10 × 10 cm^2^. The apparatus was elevated to a height of 70 cm. During the test, each animal was placed at the center of the maze facing the closed arm 60 min after administration of VP extract (50–150 mg/kg, p.o.) or vehicle. All entries to the open or closed arms were scored for 5 min, and the total time spent in each arm was recorded. An entry was defined as placing the mouse's four paws into an arm, and no time was recorded when the animal was in the center of the maze. Diazepam (0.75 mg/kg, i.p., was administered to mice 30 min before the test) was used as a reference drug.

#### 2.2.7. Inhibitory Avoidance Test (IA)

Mice were trained in a one-trial, stepping-down inhibitory avoidance paradigm (IA), a highly validated learning task in which stepping down from a platform when a context is associated with a foot shock increases step-down latency [34]. The IA training apparatus was a 50 cm × 25 cm × 25 cm Plexiglas box with a 5 cm high, 8 cm wide, and 25 cm long platform on the left end of a series of bronze bars that constitutes the floor of the box. During training, the animals were gently placed on the platform facing the left rear corner of the training box. When they stepped down and placed their four paws on the grid, they received a 2 s, 0.4 mA scrambled foot shock. They were then immediately withdrawn from the training box. Memory retention was evaluated in a test session carried out 24 h after training. In the test, trained animals were placed back in the training box platform until they eventually stepped down to the grid. The latency to step-down during the test session was taken as an indicator of memory retention. A ceiling of 180 s was imposed for the step-down latencies during the retention test. To evaluate the possible effect of the extract on the animal's memory consolidation, the treatment with HEPC (50, 100, or 150 mg/kg) or vehicle was given 30 min after the IA training session.

#### 2.2.8. Tail Suspension Test (TST) and Forced Swim Test (FST)

The TST and FST were used to assess the possible antidepressant-like effect of HEPC. The forced swim test (FST) is used to assess learned helplessness in rodents and has often been used to examine the effect of antidepressants in animal models of depression. The test was performed according to Porsolt et al. [35] with minor modifications [36]. On the day of the test, the mice were individually introduced into a glass cylinder (height: 45 cm; diameter: 20 cm) containing water (25 ± 2°C; depth: 25 cm) from which they could not escape and had to swim or float. The fluctuation time (in seconds) in the last 4 minutes of the test session was recorded to reflect the depressive-like behavior of the mice. The mice's floating status means no movement except the movement necessary to keep the mice's heads above the water to breathe. The tail suspension test (TST) is also a useful behavioral tool for examining the effects of antidepressant drugs [37]. During the test, the mice were placed in a wooden apparatus divided into 4 compartments to be acoustically and visually isolated. A metallic bar suspended from the base 50 cm above the floor was adapted to the apparatus. On this bar, the animals were suspended with adhesive tape and kept approximately 1 cm from the tip of the tail. As with the FST, immobility time was recorded during the last 4 min of a 6 min session. Imipramine (30 mg/kg, p.o), HEPC (50, 100, or 150 mg/kg, p.o.), or vehicle was administered 1 h before both tests. TST was also used to evaluate the antidepressant-like effect of HEPC when administered chronically. Imipramine (30 mg/kg, p.o.), HEPC (150 mg/kg, p.o.), or vehicle was administered daily for 15 days and, on the last day of treatment, 1 h before the test. After the experiments, the animals used in the subchronic treatment to evaluate the antidepressant-like effects of EHPC were euthanized and their brains were collected for biochemical tests.

#### 2.2.9. Time Course of the HEPC Antidepressant-Like Effect

In another set of experiments, different groups of mice were administered with HEPC 150 mg/kg (p.o.) or vehicle and subjected to the TST after 1 h, 2 h, 3 h, and 4 h from the administration. For each hour tested, a different group of animals was used, and we were careful to avoid mice learning [38].

#### 2.2.10. Euthanasia and Animal Disposal

The animals were euthanized with anesthetic overdose as decided by the UNIVALI Animal Ethics Committee (CEUA/UNIVALI), except for the group used for biochemical tests, which were euthanized by cervical dislocation to avoid possible interference in the results. The carcasses were collected and sent for disposal and incineration by responsible technicians, following laboratory and institution procedures.

### 2.3. Biochemical Analysis

The levels of gamma-aminobutyric acid (GABA) and monoamine oxidase (MAO)- A activity in the animal's brain subchronically treated for 15 days with HEPC (150 mg/kg, p.o.), imipramine (30 mg/kg, p.o.), diazepam (2 mg/kg, i.p.), or vehicle were measured. The group that received diazepam was incorporated into the protocol separately. The mice were decapitated, and the brains were quickly transferred to a cold glass plate for dissection. The brain was quickly isolated, and homogenized being stored at −80°C until further analysis, as described by Zimath et al. [38], except for samples intended for measurement of GABA levels.

#### 2.3.1. Quantification of MAO-A Enzyme Activity Levels

First, the brain homogenate was centrifuged for 10 minutes at 4000 rpm at 4°C. The supernatant was removed and centrifuged for 120 minutes at 10000 rpm at 4°C. The pellets were resuspended with phosphate buffer. Protein concentration was adjusted to 1 mg/ml and measured using bovine albumin as a standard. To evaluate the MAO-A activity, 50 *µ*L of the sample plus 30 *µ*L of 4 mM 5-HT (as a specific substrate for MAO-A) and 100 *µ*L of phosphate buffer were incubated for 20 minutes at 37°C. After 20 minutes, the reaction was stopped with 50 *µ*L of 1 M HCl. The reaction product was extracted with 2200 *µ*L of butyl acetate. The organic phase was used, and the absorbance was measured at 280 nm. To perform the blank 50 *µ*L of the sample, plus 50 *µ*L of 1 M HCl, and 100 *µ*L of phosphate buffer was incubated for 20 minutes at 37°C. Subsequently, 30 *µ*L of 4 mM 5-HT and 2200 *µ*L of butyl acetate were added. The organic phase was used and measured in a spectrophotometer at 280 nm. MAO-A enzyme activity was expressed as nmol/mg of protein [38, 39].

#### 2.3.2. Estimation of GABA by Spectrophotometry

The GABA content was determined from the whole brain homogenized in a tube containing 5 mL of 0.01 M HCl. Brain homogenate was transferred to a bottle containing 8 mL of ice-cold absolute alcohol and kept for 1 h at 0°C. The content was centrifuged for 10 min at 16,000 rpm, the supernatant was collected in a Petri dish. The precipitate was washed with 5 mL of 75% alcohol three times and washes were combined with supernatant. Next, samples were evaporated to dryness at 70°C in a water bath. To the dry mass, 1 mL water, and 2 mL chloroform were added and centrifuged at 2000 rpm. The upper phase containing GABA (2.0 mL) was separated and 10 mL of it was applied as spot on Whatman paper (N° 41). The mobile phase consisted of n-butanol (50 mL), acetic acid (12 mL), and water (60 mL). The paper chromatogram was developed with ascending technique. The paper was dried in hot air and then spread with 0.5% ninhydrin solution in 95% ethanol. The paper was dried for 1 h at 90°C. The blue color spot developed on the paper was cut and heated with 2 mL of ninhydrin solution in a water bath for 5 min. Water (5.0 mL) was added to the solution and kept for 1 h. The supernatant (2.0 mL) was decanted, and absorbance was measured at 570 nm by using spectrophotometry. GABA standard was used to extrapolate the absorbances of the samples [40].

### 2.4. Statistical Analysis

Data were evaluated for normality by the Shapiro–Wilk test and Grubbs' test was used to detect outliers. For parametric tests, the results were presented as means ± standard error of means (S.E.M). Statistical analysis was performed using one-way or two-way analysis of variance (ANOVA), when applicable, followed by the Bonferroni post-test. The data obtained in the step-down IA task were reported as median ± interquartile ranges (25 and 75). The analysis of IA data was nonparametric because this procedure involved a cutoff score, and Kruskal–Wallis's test was performed followed by Dunn's test. All analyses were performed using the GraphPad Prism 7.0 program (San Diego, USA). Values of *p* < 0.05 were considered significant.

## 3. Results

### 3.1. HEPC Effects in Animal Motor Performance of Mice Exposed to Open-Field Test (OFT)

**Figure 1:**
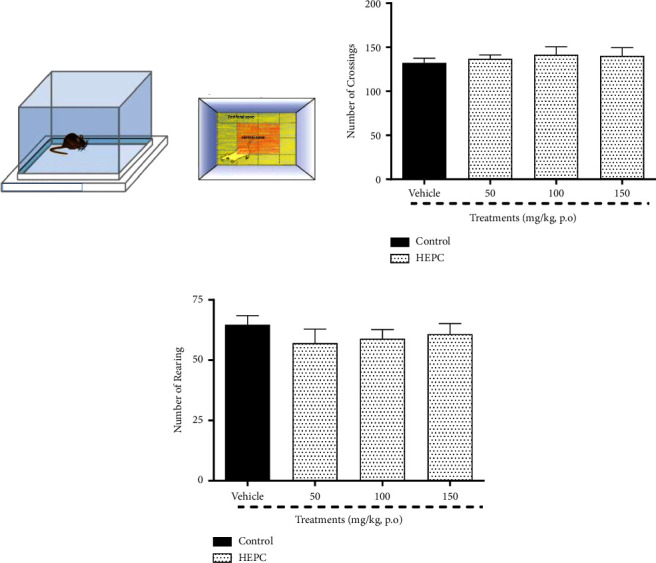
HEPC effects in animal motor performance of mice exposed to open-field test (OFT). HEPC does not change the number of mice (a) crossing or (b) rearing when submitted to the open-field test. Results are presented as the means ± S.E.M. (*n* = 8–10). ANOVA followed by Bonferroni's multiple comparisons test when compared to the vehicle group (treated with distilled water plus 2% DMSO).

No statistical difference among the experimental groups was obtained in the behavioral parameters recorded by OFT. Our data show that the administration of treatments does not change the number of animal passages (crossings), (*F*(3.36) = 0.3370, *p*=0.7986), as well as did not change the number of exploratory activities (rearing), (*F*(3.36) = 0.5570, *p*=0.6468), indicating that it probably does not change the mobility of animals in the other behavioral tests used in this study (Figures 1(a) and 1(b)).

### 3.2. HEPC Effects on Pentylenetetrazol (PTZ) and Strychnine (STR) Induced Seizure Test in Mice

**Figure 2:**
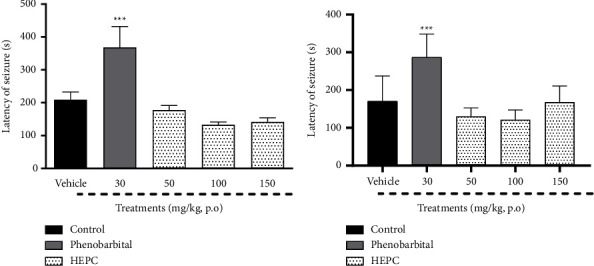
HEPC effects on Pentylenetetrazol (PTZ) and strychnine (STR) induced seizure test in mice. Phenobarbital, but not HEPC, alters latency for seizures induced by pentylenetetrazol (PTZ) (a) or strychnine (STR) (b) in mice. Results are presented as the means ± S.E.M. (*n* = 8–10). One–-way ANOVA followed by Bonferroni's multiple comparisons tests. ^*∗∗∗*^*p* < 0.0001 compared to the group treated with vehicle (treated with distilled water plus 2% DMSO).

The effect of HEPC treatment on PTZ and strychnine-induced seizure models is shown in Figure 2. In the PTZ-induced seizure (Figure 2(a)), HEPC at doses of 50, 100, or 150 mg/kg were not able to increase latency time for the onset of seizures compared with the vehicle group. As expected, phenobarbital increased the latency time for the onset of seizures (*F*(4.43) = 8.554, *p* < 0.0001). In strychnine-induced seizures (Figure 2(b)), the treatment of animals with HEPC did not increase the latency time for the onset of seizures compared to the vehicle group. Nevertheless, in this test, the typical anticonvulsant effect of phenobarbital was verified (*F*(4.42) = 17.94, *p* < 0.0001).

### 3.3. HEPC Effects on Pentobarbital-Induced Hypnosis (PIH) in Mice

**Figure 3:**
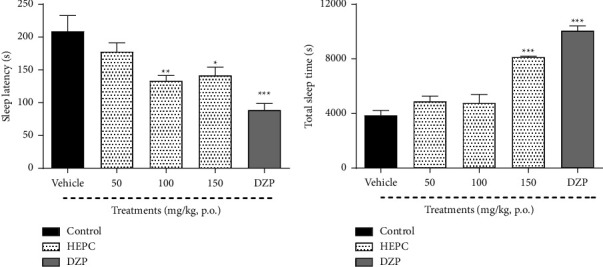
HEPC effects on pentobarbital-induced hypnosis (PIH) in mice. HEPC increases sleep latency (a) and total sleep time (b) in pentobarbital sleep induction test in mice. Results are presented as the means ± S.E.M. (*n* = 6–8), one-way ANOVA followed by Bonferroni post-test. ^*∗∗∗*^*p* < 0.0001, ^*∗∗*^*p* < 0.01, and ^*∗*^*p* < 0.05 compared to the group treated with vehicle (treated with distilled water plus 2% DMSO). DZP-diazepam.

The effect of HEPC treatment on pentobarbital-induced hypnosis is shown in Figure 3. The results obtained demonstrate that the treatment of animals with HEPC at 100 or 150 mg/kg decreased the sleep latency time when compared with the vehicle group (*F*(4.43) = 9.501, *p* < 0.0001). Diazepam, used as a positive control, also promoted a decrease in sleep latency time when compared to the vehicle group (*p* < 0.001), (Figure 3(a)).

In Figure 3(b), the total sleep time was shown, which was changed only with the treatment of HEPC at 150 mg/kg, when compared with the vehicle group (*F*(4.44) = 42.14, *p* < 0.001). The treatment of animals with diazepam showed similar results, promoting an increase in total sleep time, and reproducing its characteristic hypnotic effect (*p* < 0.001).

### 3.4. HEPC Effects on Anxious Behavior of Mice Exposed to Elevated Plus-Maze Test (EPM)

**Figure 4:**
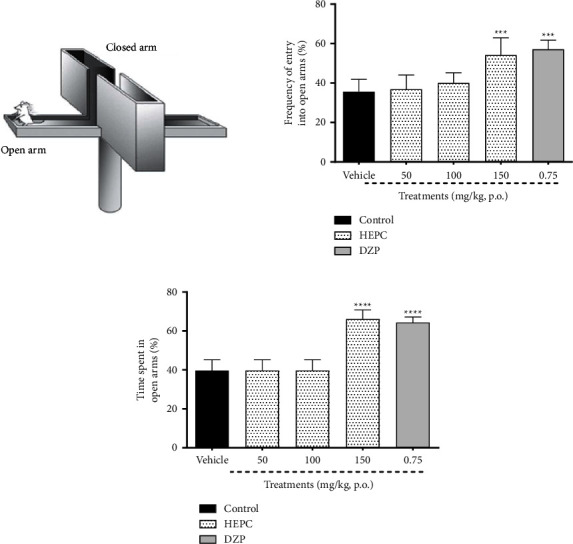
HEPC effects on anxious behavior of mice exposed to elevated plus-maze test (EPM). HEPC administration increased the mice frequency of entries (a) and time spent (b) in the open arms of the apparatus. Results are presented as the means ± S.E.M. (*n* = 8–10). One-way ANOVA was followed by Bonferroni's post-test. ^*∗∗∗*^*p* < 0.001 and ^*∗∗∗∗*^*p* < 0.0001 compared to the group treated with vehicle (VEH). DZP- diazepam.

The results presented in Figure 4 demonstrate that the treatment of the animals with HEPC only at 150 mg/kg increased the frequency of entry into open arms (Figure 4(a)), and the time of mice exploration in these arms (Figure 4(b)) of the apparatus, when compared with the vehicle group ((*F*(4.43) = 23.64, *p* < 0.0001), (*F*(4.43) = 74.78, *p* < 0.0001), respectively). As expected, during the experiment, the classic anxiolytic-like effect of diazepam was observed, increasing such behavioral parameters in the open arms of the EPM when compared to the vehicle group (*p* < 0.001).

### 3.5. HEPC Effects on Mice Submitted to Inhibitory Avoidance Test (IAT)

**Figure 5:**
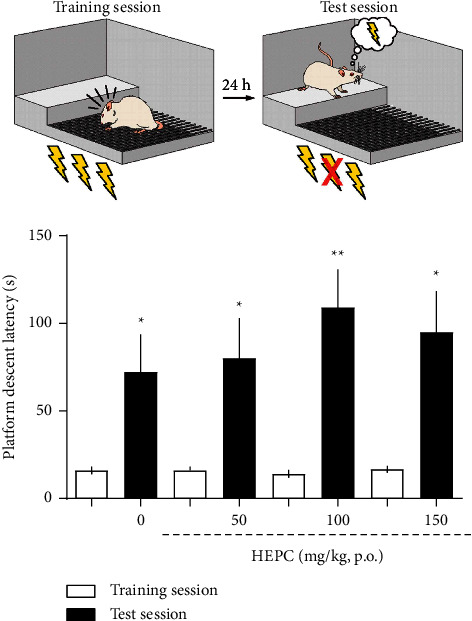
HEPC effects on mice submitted to inhibitory avoidance test (IAT). HEPC does not alter the memory of animals submitted to the inhibitory avoidance test. The white bars refer to the results obtained in the training sessions, and the dark bars refer to the results of the test session. The treatments were given immediately after training in a single trial, on memory retention of inhibitory avoidance measured 24 h later. Each bar represents the median (interquartile range) for 8 to 10 animals per group. The data obtained in the step-down IA task were reported as median ± interquartile ranges (25 and 75). The analysis of IA data was nonparametric because this procedure involved a cutoff score, and the Kruskal–Wallis test was performed followed by Mann–Whitney's *U* test. ^*∗*^*p* < 0.05 and ^*∗∗*^*p* < 0.01.

Figure 5 shows the effects of HEPC on memory retention in animals submitted to IAT. It was observed that the animals learned the inhibitory avoidance task, comparing the test and training sections for each treatment group, with an increase in the latency of descent from the apparatus platform. However, when the test session is evaluated (only the dark bars in Figure 5) and compared with the vehicle (represented by the 0 doses of HEPC), it is verified that there was no statistically significant effect of the treatment on the animals' memory, demonstrating thus, that HEPC did not exhibit effects on the memory of the animals at the doses tested.

### 3.6. HEPC Antidepressant-Like Effect in Mice Submitted to Tail Suspension Test (TST) and Forced Swim Test (FST)

**Figure 6:**
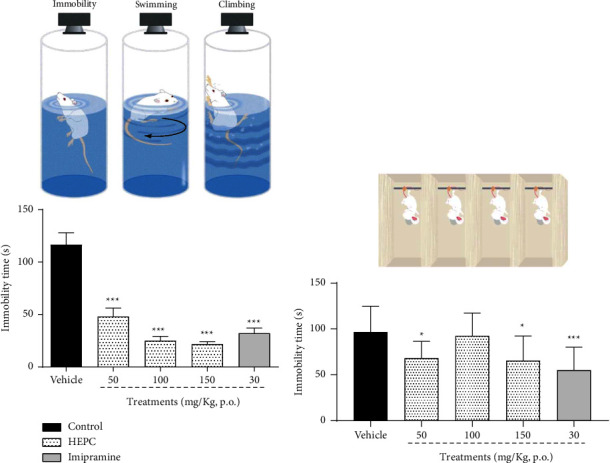
HEPC antidepressant-like effect in mice submitted to tail suspension test (TST) and forced swim test (FST). HEPC elicited an antidepressant-like effect in mice submitted to the forced (a) swimming test and in the tail (b) suspension test. Results are presented as the means ± S.E.M. (*n* = 8–10). One-way ANOVA was followed by Bonferroni's post-test. ^*∗∗∗*^*p* < 0.0001 and ^*∗*^*p* < 0.05 compared to the group treated with vehicle (VEH).

**Figure 7:**
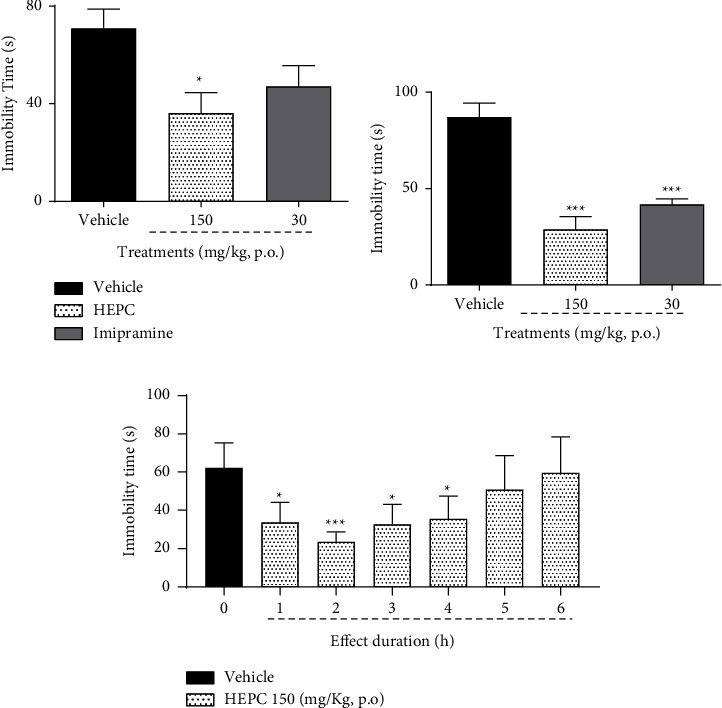
Time course of the HEPC antidepressant-like effect in mice subchronically treated. Effect of HEPC in the immobility time of mice submitted to the tail suspension test (a) 7 days and (b) 14 days after treatments. Panel (c) represents the time course of the HEPC antidepressant-like effect at 150 mg/kg (p.o.). Results are expressed as mean ± S.E.M (*n* = 8–10). ^*∗*^*p* < 0.05 and ^*∗∗∗*^*p* < 0.0001 compared with the vehicle-treated group; one-way ANOVA followed by Bonferroni's post-test.

The antidepressant-like property of HEPC is demonstrated in Figures 6(a) and 6(b). In Figure 6(a), it is observed that the acute treatment of the animals with HEPC at doses of 50, 100, and 150 mg/kg promoted a significant decrease in the immobility time of the animals when submitted to FST when compared with the control group treated with vehicle ((*F*(4.38) = 31.71, *p* < 0.0001)). In the TST (Figure 6(b)), only HEPC at 50 and 100 mg/kg were effective in reducing the animals' immobility time (*p* < 0.001) when compared to the vehicle-treated control group ((*F*(4.38) = 4.671, *p*=0.0036)). In both tests, respectively, the classic antidepressant-like effect of imipramine was observed (*p* < 0.001) compared to the vehicle-treated control group. The results observed in Figures 7(a) and 7(b), respectively, demonstrate that after 7 and 14 days of treatment, HEPC (150 mg/kg) continues to promote an antidepressant-like effect, evaluated by the decrease in the immobility time of mice when submitted to FST when compared to the vehicle-treated control group (*p* < 0.0001). In this experiment, the antidepressant-like effect of imipramine (30 mg/kg) was observed only on the 14th day when compared to the vehicle-treated control group ((*F*(2.23) = 24.84, *p* < 0.0001); (*F*(2.23) = 4.585, *p*=0.0122), respectively). Regarding the duration of the antidepressant-like effect of HEPC, it was observed (Figure 7(c)) that the treatment with HEPC (150 mg/kg, p.o.) produced a significant effect by reducing the immobility time of the animals after 1, 2, 3, and 4 hours (*p* < 0.05, *p* < 0.001, *p* < 0.05, respectively) of its administration when compared with the vehicle-treated control group. The peak of the antidepressant-like effect was observed 2 h after applying HEPC ((*F*(6.51) = 10.64, *p* < 0.0001)). In the experiments related to the subchronic treatment with HEPC, as well as the time of action of the same, the animals were also submitted to the open-field test without any change in their motor performance (results not shown).

### 3.7. HEPC Effects on MAO-A Enzyme Activity and the Estimation of GABA by Spectrophotometry Measured in Mice's Brain

**Figure 8:**
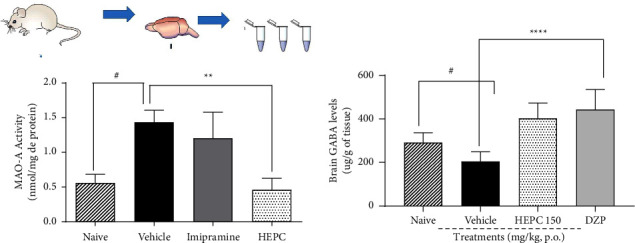
HEPC effects on MAO-A enzyme activity and the estimation of GABA by spectrophotometry measured in mice's brain. HEPC decreased MAO-A activity (a) and increased GABA brain levels (b). ^*∗∗*^*p* < 0.001 and ^*∗∗∗∗*^*p* < 0.0001 compared to vehicle. ^#^*p* < 0.05, compared to Naive. Results are presented as the means ± S.E.M. (*n* = 5–10). One-way ANOVA was followed by Bonferroni's post-test. Naïve = animals without treatment.

To relate a possible antidepressant mechanism of action elicited by HEPC, the effects of treatments on the activity of the MAO-A enzyme were analyzed. Figure 8(a) shows that HEPC promoted a decrease in MAO-A activity (*p* < 0.01), when compared to the vehicle group ((*F*(3.20) = 6.388, *p*=0.0068)). On the other hand, the effects obtained with HEPC on the estimation of GABA revels that the treatment with HEPC and DZP promoted a significant increase in the levels of GABA when compared with the vehicle (*p* < 0.05), as demonstrated in the Figure 8(b) ((*F*(3.20) = 16.10, *p* < 0.0001)).

## 4. Discussion

The investigation of new pharmacological strategies for the treatment of mood disorders, sleep disorders, or anxiety disorders is essential for the discovery of drugs with efficacy, fewer side effects, and low toxicity which could be used in longer treatments, and medicinal plants play an important role in this context [11, 12, 41]. Humanity has explored plants' psychoactive effects for millennia, using them in their most natural form [42, 43]. However, the pharmaceutical market has been demanding the pharmacological validation of extracts and/or active principles, their efficacy, and above all, security regardless of whether the use is for nervous system pathologies [44]. Therefore, to contribute to this field, this research investigated the psychoactive effect of the hydroalcoholic extract of *Piper cernuum* (HEPC), using experimental tools employed to screen psychotropic drugs.

Based on the literature on the pharmacological potential of the *Piper* genus on epilepsies, we investigated the effect of HEPC in two chemical models of seizures. The pentylenetetrazol- and the strychnine-induced seizure model [45]. Moreover, piperine, a marker of the genus, exhibits an antiepileptic effect detected in these models and in others [46, 47]. Essential oils obtained from *Piper*'s species also exhibit anti-convulsant properties [48]. However, neither the treatment with the essential oil of the plant (results not shown) nor the treatment with HEPC, at the doses used, administered acutely, did not demonstrate an anticonvulsant effect in both seizure tests. To observe an effect with subchronic treatment (15 days) at the highest dose, animals were subjected to a pentylenetetrazol-induced seizure model (results not shown), and even then, the effect was not detected.

Sleep disorders are frequent in neuropsychiatric and/or neurodegenerative diseases. The use of herbs with hypnotic characteristics in the management of insomnia is common among people and, herbal formulations with renowned hypnotic effects are easy to find [49, 50]. A plant with multiple effects can be advantageous in treating neuropsychiatric diseases whose patients exhibit sleep disorders [51]. In the present study, we evaluated the hypnotic effect of HEPC, and mice treated with the extract were subjected to the barbiturate-induced sleep test.

Substances that depress the CNS, in general, increase the duration of sleep produced by pentobarbital, and there may also be a reduction in the latency for the effect of the barbiturate used, seen by the reduction in the induction time, that is, from the application of the barbiturate until the loss of the postural reflex by the animal [52]. Our results showed that HEPC effectively decreased sleep latency and increased the total sleep time of animals submitted to the barbiturate-induced sleep test. It is expected that an agent with allosteric effects on the GABAA receptor promotes pentobarbital potentiation and, therefore, a depressant effect on the CNS [53] and that hypnotic substance reduce sleep latency and increase the total sleep time of the animals submitted to barbiturate-induced sleep test [32]. Therefore, our results corroborate those that evidenced the hypnotic effects of *Piper* sp. on sleep-related disorders, mainly regarding their maintenance [54].

The most widespread psychoactive effect of the *Piper* genus in the literature is, without a doubt, the anxiolytic effect. Many species such as *Piper nigrum* and *Piper sylvaticum* Roxb. have this pharmacological property very well documented and studied [55]. However, undoubtedly, the most studied species of the genus regarding this pharmacological property is *Piper methysticum* or simply Kava Kava [14]. There are several articles evidencing the use of Kava for generalized anxiety disorders [56].

Based on it, the anxiolytic effect of HEPC was tested in animals subjected to the elevated plus-maze test (EPM), an experimental tool frequently used to detect and evaluate the anxiolytic/anxiogenic properties of drugs. The entries and time spent in the open arms is the main indicator of fear in the EPM, given that an open area is extremely aversive to rodents. In addition, no thigmotaxis enhances the state of fear and aversion in the animals in open arms [33]. Our results with HEPC showed that only the treatment with a dose of 150 mg/kg was effective in increasing the frequency of entry into the open arms of the apparatus, as well as increasing the time spent by the animals in the same arm, suggesting a type-anxiolytic effect of this extract, making *Piper cernuum* an interesting source for research to new therapeutic option in the management of anxiety.

The effect of HEPC on memory consolidation was also examined, and effects on the animals' memory were not observed in the inhibitory avoidance test. Anxiolytic and hypnotic substances that do not promote cognitive impairment are welcome from a pharmacological point of view. BDZs are still the most used pharmacological class to produce sedative and hypnotic activities, but they have a propensity effect to cause cognitive impairment [57].

Mice acutely administered with HEPC presented a reduction in the immobility time when exposed to the FST and TST, both predictive behavioral paradigms to evaluate the antidepressant-like effect of substances in rodents [35, 37, 58]. Additionally, we verified that the antidepressant-like effect of HEPC also occurs with subchronic treatment since the immobility time of the animals in the FST at 7 and 14 days of treatment was reduced. Noteworthy, HEPC exerted an antidepressant-like effect for 4 hours from the administration, without compromising the locomotor activity of mice when exposed to the OFT. Crossings number in the OFT was registered as a parameter to investigate possible effects triggered by the treatments on the motor activity of mice, to discard a false positive or negative result from TST and FST, as well as in other behavioral tests performed here that require the integrity of the animals' motor system [33, 38].

The relationship between *Piper* and depression is little explored in the literature, compared to insomnia [59], epilepsy [54], and anxiety [41]. The articles are more related to piperine and its role as an antidepressant agent [60]. In recent times we have studied the pharmacological potential of *Piper amplum*, *Piper aduncum* L, and *Piper molluscum* as psychotherapeutic agents and we are having positive results regarding antidepressant-like effects (work in progress). Thus, the focus of the present study was to investigate the antidepressant-like effect of HEPC.

The mechanisms by which HEPC could be acting were partially investigated in this study. However, further studies are needed to clarify better the pharmacodynamic mechanism of action of the plant extract, which may be one of the limitations of the present study that can be remedied in later studies. On the other hand, we tried through the biochemical assays available in our laboratories to study two systems related to anxiety and depression.

The antidepressant and anxiolytic-like effects of the extract were observed in behavioral trials with acute and subchronic treatments (mainly regarding the antidepressant effect). Thus, animals treated with repeated doses of the extract had decreased depressive-like compounds and they were used in biochemical tests. So, the participation of the GABAergic system in the extract's mechanism of action was investigated in the animals' brains. The mechanisms underlying the pathophysiology and treatment of depression and stress-related disorders remain unclear, but studies in depressed patients and rodent models are beginning to yield promising insights [61]. The implication of GABA is based mainly on animal models, whereas clinical studies in depressed patients show alterations in GABA levels in plasma and cerebrospinal fluid. Neuroimaging studies using spectroscopy also indicate decreased GABA levels in different brain areas, which may normalize after antidepressant therapy, and these findings translate into clinical response [62, 63]. On the other hand, the literature is rich on the relationship between stress and depression [64], showing this relationship in rodents and humans [65]. The period of stress can result in the chronicity of depression, and the literature has shown that even in rodents, subchronic stress induces similar behavioral effects in male and female mice despite sex-specific molecular adaptations in the *nucleus accumbens* [66]. In addition, some studies have shown that the use of the depression model induced by chronic or subchronic stress increases oxidative stress or changes neurotransmitter levels in important mesolimbic structures in the brains of animals treated with the vehicle when compared to the naive group and that the treatment of animals with antidepressants, especially inhibitors selective serotonin and norepinephrine reuptake or substances of natural origin, ameliorates stress-induced behavioral as well as oxidative alterations [67, 68]. Our results demonstrated that the stress generated by the subchronic administrations and daily handling of animals can promote in the groups treated with vehicle an increase in MAO activity and a decrease in the levels of GABA in the brain of the animals, different from that observed in naive animals. The results showed that there is a significant increase in GABA levels in the groups treated with HEPC, supporting the idea that HEPC interacts with the GABAergic system in terms of increasing its neurotransmission. GABA is the most abundant inhibitory neurotransmitter in the CNS [69], exerting its effects on GABAA, GABAB, and GABAC receptors [70–73]. Given that the GABAergic system is closely linked with the regulation of sleep [74], anxiety [75], and epilepsy [76], it is possible to infer that, at least in part, the anxiolytic and hypnotic properties of the extract involve this system.

The relationship between the GABAergic system with depression is not yet well investigated in the literature, although it exists [61], perhaps because there are no antidepressant drugs that act directly on the GABAergic system. However, it is known that the glutamatergic system, which is controlled by the GABAergic system, is involved with the pathogenesis of depression. The glutamatergic hypothesis of depression is an exacerbation of the glutamatergic system that is observed in depressive patients [77], suggesting an indirect involvement of this system with depressive episodes [78].

In addition, studies have been demonstrating that depression and chronic stress exposure cause atrophy of neurons in cortical and limbic brain regions implicated in depression, and brain imaging studies have been demonstrating altered connectivity and network function in the brains of depressed patients. Studies of the neurobiological basis of these alterations have focused on an excitatory glutamate neuron together with an inhibitory GABA interneuron. They demonstrate structural, functional, and neurochemical deficits in both major neuronal types that could lead to signal integrity degradation in cortical and hippocampal regions [61]. Therefore, our results regarding the extract's antidepressant-like properties may also be related to the increase in GABA levels in the brain of the treated animals.

Antidepressants may act by modulating the MAO enzyme, and this mechanism has already been detected in secondary metabolites from plants [79]. The MAO enzyme metabolizes xenobiotics and endogenous amines and neurotransmitters, including 5-hydroxytryptamine (5-HT, serotonin), dopamine (DA), noradrenaline (NA), tyramine, and tryptamine [80]. It occurs as two isoenzymes, MAO-B is involved in neurodegenerative diseases and MAO-A in psychiatric conditions such as MDD [81]. Here, the pretreatment with HEPC promoted the MAO-A inhibition in the brain, maybe in structures that are closely related to the limbic system and mood modulation. Together, these results indicate that MAO-A inhibition may be involved in the HEPC antidepressant and anxiolytic effects. Inhibition of MAO-A by the extract would consequently promote an increase in the brain concentration of important neurotransmitters, such as serotonin, whose alterations in its levels are involved in both diseases, depression, and anxiety.

The relationship between the pharmacological effects detected in this study and the phytochemical profile of the extract used can be analyzed. As reported previously, *P. cernuum* leaves are used in folk medicine as an infusion or a macerate with alcohol, and therefore the alcoholic extract was chosen to access the psychopharmacology properties of this plant in this study. Wolff and collaborators [31] described that this same extract did not present significant toxicity when administered to male or female rats and presented a high-performance liquid chromatography profile for this extract. In the leaves of *P. cernuum* were found cinnamic acids derivatives, lignans as cubebin and hinokinin [18, 20], which are extracted more efficiently with ethanol and can be present in HEPC. Some of these phytoconstituents exhibit psychopharmacological properties already reported in the literature and may be contributing to the psychoactive effects found in *P. cernuum*. For cinnamic acid, antidepressant effects have been recently reported [82, 83], as well as the anticonvulsant potential of this compound and its derivatives [84]. GABA transporters serve as a target for anxiolytic, antidepressant, and antiepileptic therapies. Interaction with important neurotransmitter transporters has been characterized for a lignan derivative (−)-cubebin, and (−)-hinokinin, and the results obtained to date suggest that hinokinin can serve as a tool to develop new therapeutic anxiety drugs that target dopamine, norepinephrine, and GABA transporters [85]. Another interesting class in Piperaceae was alkaloids. This class of compounds has psychoactive characteristics that are also interesting and may be directly or indirectly involved in many of its effects. In the extract used, the alkaloids were not individually characterized, however, these phytoconstituents have already been identified in several species of pipers being responsible for their various effects [86, 87].

## 5. Conclusion

Together, the results suggest that HEPC has therapeutic potential as an antidepressant, anxiolytic, and sedative-hypnotic agent. Preliminarily, is possible to suggest that the neuropharmacological effects of HEPC could be, at least in part, related to the modulation of the GABAergic system and/or MAO-A activity. However, studies are needed to evaluate the chronic effects of HEPC on anxiety to verify if the increase in GABA levels could also be related to its anxiolytic effect. We also suggest that further experiments be conducted with male mice to verify possible variations in the pharmacological effects of the extract according to sex.

## Data Availability

The data that support the findings of this study are available from the corresponding author, MMS, upon reasonable request.
